# Gram-Scale Synthesis of (*R*)*-P-*Chlorophenyl-1,2-Ethanediol at High Concentration by a Pair of Epoxide Hydrolases

**DOI:** 10.3389/fbioe.2022.824300

**Published:** 2022-02-28

**Authors:** Dong Zhang, Yuqing Lei, Tingting Wang, Wenqian Lin, Xingyi Chen, Minchen Wu

**Affiliations:** ^1^ Key Laboratory of Carbohydrate Chemistry and Biotechnology, Ministry of Education, School of Biotechnology, Jiangnan University, Wuxi, China; ^2^ Yancheng Juheng Road Primary School, Yancheng, China; ^3^ Wuxi School of Medicine, Jiangnan University, Wuxi, China

**Keywords:** bi-enzymatic catalysis, kinetic resolution, enantioconvergent hydrolysis, epoxide hydrolase, high substrate concentration

## Abstract

(*R*)-*p*-chlorophenyl-1,2-ethanediol (*p*CPED) is an important intermediate for the synthesis of (*R*)-eliprodil that is widely applied in the treatment of ischemic stroke. To prepare (*R*)-*p*CPED with high enantiomeric excess (*ee*_p_) and yield via the enantioconvergent hydrolysis of racemic styrene oxide (*rac-p*CSO) at high concentration, the bi-enzymatic catalysis was designed and investigated by a pair of epoxide hydrolases, a mutant (*Pv*EH1^Z4X4-59^) of *Phaseolus vulgaris* EH1 and a mutant (*Rp*EH^F361V^) of *Rhodotorula paludigena Rp*EH*.* Firstly, the maximum allowable concentration of *rac*-*p*CSO was confirmed. Subsequently, the addition mode and the weight ratio of two *Escherichia coli* cells were optimized. Finally, under the optimized reaction conditions—the cell weight ratio 20:1 of *E. coli*/*pveh*1^z4x4-59^ to *E. coli/rpeh* ^F361V^, a simultaneous addition mode, and reaction temperature at 25°C—300 mM *rac-p*CSO in the 100 ml 4% (v/v) Tween-20/phosphate buffer system (100 mM, pH 7.0) was completely hydrolyzed within 5 h, affording (*R*)-*p*CPED with 87.8% *ee*_p_, 93.4% yield, and 8.63 g/L/h space–time yield (STY). This work would be an efficient technical strategy for the preparation of chiral vicinal diols at industrial scale.

## Introduction

Highly value-added chiral chemicals, such as epoxides and/or their corresponding vicinal diols, are versatile chiral building blocks applied in pharmaceuticals, fine chemicals, and agrochemical industries due to the fact that they can perform various chemical reactions with nucleophiles, electrophiles, acids, and bases (Zou et al., 2018). For example, (*R*)-*p*CSO is an important intermediate for the synthesis of rimonabant, an antagonist of the cannabinoid CB1 receptor (CB1R), and (*R*)-*p*-chlorophenyl-1,2-ethanediol (*p*CPED) for the synthesis of (*R*)-eliprodil, a quite promising neuroprotective agent applied in the treatment of ischemic stroke (Di Fabio et al., 1995; Saini and Sareen, 2017). Interestingly, it has been demonstrated that the biological activity of eliprodil *in vivo* is essentially associated with its (*R*)-(−)-enantiomer rather than its (*S*)-(+)-enantiomer and that the pharmacodynamic properties of the two enantiomers are different (Di Fabio et al., 1995). With increasing environment awareness, the biocatalysis using the whole cells or enzymes, an environmental-friendly process with high stereoselectivity and little or no byproducts, has attracted much attention in recent years (Kotik et al., 2013; Patel, 2018). Epoxide hydrolases (EHs, EC 3.3.2.-), which widely exist in microorganisms, plants, invertebrates, and mammals, can stereoselectively catalyze the opening of an active three-membered oxirane ring of racemic epoxides, retaining epoxide enantiomers and/or producing enantiopure vicinal diols (Woo et al., 2015). Based on the catalytic mechanisms of the given EH–epoxide pairs, the asymmetric hydrolysis of *rac*-epoxides can be divided into two pathways: kinetic resolution and enantioconvergent hydrolysis (Bala and Chimni, 2010). Compared with the kinetic resolution having an intrinsic limitation of 50% maximum yield of epoxide enantiomers, the enantioconvergent hydrolysis can completely convert *rac*-epoxides into chiral vicinal diols with up to 100% theoretical yield (Wu et al., 2015).

The enantioconvergent hydrolysis of *rac*-epoxides by mono-enzymatic catalysis is an ideal process for preparing desired chiral diols, whereas few EHs have high and complementary regioselectivities on (*S*)- and (*R*)-enantiomers leading to high enantiomeric excess values of chiral diols (*ee*_p_) (Li et al., 2019a). As numerous EHs with enantio- and/or regio-selectivity have been characterized, bi-enzymatic catalysis has been designed to increase the concentration of epoxides and productivity of chiral diols with high *ee*_p_ by screening pairs of EHs and reaction systems, and optimizing reaction conditions (Hu et al., 2016). For example, using the immobilized *St*EH with the immobilized *An*EH possessing complementary enantio- and regio-selectivity in a sequential addition mode, 4 mM *rac-p*CSO was completely and convergently converted into (*R*)*-p*CPED with 89% *ee*_p_ in 2.65 h (Karboune et al., 2005). However, the low catalytic efficiency and substrate or product inhibition limited the practical application of EHs (Deregnaucourt et al., 2007). Therefore, to break through these bottlenecks, one of the effective strategies is to screen pairs of EHs and to improve their catalytic performance.

In our previous studies, a *Pv*EH1-encoding gene (*pveh*1, GenBank accession no: KR604729) was cloned and expressed in *Escherichia coli* BL21(DE3) (Ye et al., 2016). Then, its mutant *Pv*EH1^Z4X4-59^ towards *rac*-*p*CSO was studied. The catalytic performance analysis indicated that *Pv*EH1^Z4X4-59^, exhibiting high preference towards (*S*)-*p*CSO and moderate complementary regioselectivity towards (*S*)-*p*CSO (α_*S*_ = 94.5%) and (*R*)-*p*CSO (β_*R*_ = 80%), can catalyze the hydrolysis of *rac-p*CSO (10 mM) in an enantioconvergent way, affording (*R*)-*p*CPED with 83.3% *ee*_p_ at 100% conversion ratio (*c*) in 24 h. However, its unsatisfactory regioselectivity coefficient β_*R*_ (80%) led to low *ee*_p_ of (*R*)-*p*CPED, while low substrate concentration led to low catalytic efficiency. In this work, by screening our “in house” available epoxide hydrolases, *Rp*EH^F361V^, a single site mutant of *Rp*EH from *Rhodotorula paludigena*, was selected, which was enantio-complementary to *Pv*EH1^Z4X4-59^. *Rp*EH^F361V^, preferentially hydrolyzing the (*R*)-*p*CSO with a main attack at a β-carbon atom (β_*R*_ = 93%), can enantioselectively hydrolyze *rac*-*p*CSO at high concentration (800 mM), affording (*R*)-*p*CPED and retaining (*S*)-*p*CSO with over 99% *ee*_s_ and 43.2% yield at 55.5% *c* in 12 h. Then, the bi-enzymatic catalysis by using the two complementary EHs of *Pv*EH1^Z4X4-59^ and *Rp*EH^F361V^ was designed for the enantioconvergent hydrolysis of *rac*-*p*CSO at high concentration. Additionally, the addition mode and the weight ratio of two recombinant *E. coli* cells expressing *Pv*EH1^Z4X4-59^ and *Rp*EH^F361V^, respectively, were optimized to prepare (*R*)-*p*CPED with high *ee*_p_, yield, and space–time yield (STY).

## Materials and Methods

### Plasmids, Strains, and Chemicals

Both recombinant plasmids (pET-28a-*pveh*1^z4x4-59^ and pET-28a-*rpeh* ^F361V^) and EH-expressing *E. coli* transformants (*E. coli*/*pveh*1^z4x4-59^ and *E. coli*/*rpeh* ^F361V^) were constructed and preserved in our lab (Hu et al., 2020b; Xu et al., 2020). The transformants were grown in Luria–Bertani (LB) medium and induced by isopropyl-β-D-thiogalactoside (IPTG). *Rac-p*CSO, (*S*)*-p*CPED, and (*R*)*-p*CPED (Energy, Shanghai, China) were used for the assays of activities and regioselectivity coefficients of EHs. All other chemicals were of analytical grade.

### EH Expression of *E. Coli* Transformants and EH Activity Assay

Single colonies of *E. coli* transformants, such as *E. coli/pveh*1^z4x4-59^ and*/rpeh* ^F361V^ were separately inoculated into the LB medium containing 100 μg/ml kanamycin and cultured at 37°C for 12–14 h as the seed culture. Then, 1% (v/v) seed culture was inoculated into the fresh LB medium and cultured for 3–4 h until the OD_600_ value reached 0.6–0.8. After inducing with 0.05 mM IPTG at 20°C for 10 h, the *E. coli* transformant cells were harvested by centrifugation (8,000 rpm for 5 min, 4°C) and resuspended in 100 mM Na_2_HPO_4_-NaH_2_PO_4_ buffer (pH 7.0) to a fixed concentration of 100 mg wet cells/ml with nearly 83.3% moisture content of the wet cells, unless stated otherwise. The cell suspension was used as the biocatalyst. Comparatively, *E. coli* BL21(DE3) transformed with pET-28a, designated *E. coli*/pET-28a, was used as the negative control.

The catalytic activities of *Pv*EH1^Z4X4-59^ and *Rp*EH^F361V^ towards *rac*-*p*CSO were measured as previously described (Li et al., 2019b), with slight modification. In detail, 100 μl cell suspension was mixed with 25 μl 200 mM *rac*-*p*CSO dissolved in methanol (at a final concentration of 10 mM) and 375 μl 100 mM Na_2_HPO_4_-NaH_2_PO_4_ buffer (pH 7.0), incubated at 25°C for 10 min, and terminated by the addition of 2 ml methanol. The reaction sample was analyzed by high-performance liquid chromatography (HPLC), using a Waters e2695 apparatus (Waters, Milford, MA) equipped with an XBridge BEH C18 column. The mobile phase of methanol/H_2_O (7:3, v/v) was used at a flow rate of 0.8 ml/min and monitored using a 2489 UV–Vis detector at 220 nm. One activity unit (U) of *Pv*EH1^Z4X4-59^ or *Rp*EH^F361V^ was defined as the mount of whole cells of *E. coli/pveh*1^z4x4-59^ or*/rpeh* ^F361V^ hydrolyzing 1 μmol *rac*-*p*CSO per minute under the given assay conditions (at pH 7.0 and 25°C for 10 min).

### Regioselectivity Coefficients Assay

The EHs regioselectivity coefficients, α_*S*_ (or β_*S*_ = 1 − α_*S*_) and β_*R*_ (or α_*R*_ = 1 − β_*R*_), were applied to evaluate the probabilities attacking on C_α_ (a more hindered benzylic carbon in the oxirane ring) of (*S*)-enantiomer and on C_β_ (a less hindered terminal carbon) of (*R*)-enantiomer, respectively. In this study, the regioselectivity assay was carried out as follows: 100 μl cell suspension was mixed with 50 μl 200 mM *rac*-*p*CSO and 850 μl 100 mM Na_2_HPO_4_-NaH_2_PO_4_ buffer (pH 7.0) and incubated at 25°C. During the hydrolytic process, aliquots of 50 μl reaction samples were drawn out, extracted with 1 ml ethyl acetate and assayed by HPLC equipped with an AS-H column (Daicel, Osaka, Japan) under the same assay conditions as described above, except for the mobile phase n-hexane/isopropanol (8:2, v/v). The conversion ratio (*c* value) of *rac*-*p*CSO was defined as the percentage of its consumed amount to initial one, while the yield of (*R*)-*p*CPED was referred as the percentage of its generated amount to initial amount of *rac*-*p*CSO. The *ee*_s_ of (*S*)-*p*CSO and *ee*_p_ of (*R*)-pCPED were calculated according to the following equations: *ee*_s_ = [(*S*_s_ − *R*_s_)/(*R*_s_ + *S*_s_)] × 100% and *ee*_p_ = [(*R*_p_ − *S*_p_)/(*R*_p_ + *S*_p_)] × 100%, in which *R*_s_ and *S*_s_ represent the concentrations of (*R*)- and (*S*)-*p*CSO, respectively, and *R*_p_ and *S*_p_ the concentrations of (*R*)- and (*S*)-*p*CPED (Li et al., 2019c). Additionally, the “final *ee*_p_” was defined as *ee*_p_ calculated from the formula above at 100% *c* value. The α_*S*_ and β_*R*_ values of *Pv*EH1^Z4X4-59^ and *Rp*EH^F361V^ towards (*S*)- and (*R*)-*p*CSO can be obtained by linear regression: *ee*_p_ = (α_*S*_ + β_*R*_ − 1) + [(β_*R*_ − α_*S*_) × *ee*_s_ × (1 − *c*)/*c*].

### Enantioconvergent Hydrolysis of *rac-p*CSO at Elevated Concentrations by *E. Coli*/*pveh*1^z4x4-59^

The enantioconvergent hydrolytic reactions, in the final volume of 2 ml 4% (v/v) Tween-20/phosphate buffer system (100 mM, pH 7.0), of *rac*-*p*CSO at concentrations ranging from 50 to 250 mM, were carried out, respectively, at 25°C using 200 mg/ml wet cells of *E. coli*/*pveh*1^z4x4-59^. Aliquots of 100 μl reaction samples were drawn out at 12 and 24 h and analyzed by HPLC under the same conditions as described above. Using the *c* and *ee*_p_ values as the criteria, the maximum allowable concentration (MAC) of *rac*-*p*CSO was confirmed.

### Scale-Up Kinetic Resolution of *rac-p*CSO by *E. Coli*/*rpeh* ^F361V^

The enantioselective hydrolytic reactions, in seven aliquots of 2 ml 4% (v/v) Tween-20/phosphate buffer (100 mM, pH 7.0) system consisting of 40 mg/ml wet cells of *E. coli*/*rpeh* ^F361V^ and *rac*-*p*CSO at elevated concentrations from 300 to 900 mM, were conducted, respectively, at 25°C for 12 h and analyzed by chiral HPLC. Using the *c* value of *rac*-*p*CSO and *ee*_s_ value of (*S*)-*p*CSO as the criteria, the MAC of *rac*-*p*CSO was confirmed. The gram-scale kinetic resolution of *rac*-*p*CSO at MAC in the 30 ml Tween-20/phosphate buffer system was conducted until the *ee*_s_ of (*S*)-*p*CSO reached over 99%.

### Enantioconvergent Hydrolysis of *rac-p*CSO at Elevated Concentrations by *E. Coli*/*pve*h1^z4x4-59^ and *E. Coli*/*rpeh* ^F361V^

Using the *c* and *ee*_p_ values as the criteria, enantioconvergent hydrolytic reations of *rac*-*p*CSO at concentrations ranging from 100 to 800 mM were carried out in the 2 ml 4% (v/v) Tween-20/phosphate buffer system (100 mM, pH 7.0), using 200 mg/ml wet cells of *E. coli*/*pveh*1^z4x4-59^ and 40 mg/ml wet cells of *E. coli*/*rpeh* ^F361V^ at 25°C for 12 h to conform the MAC of *rac*-*p*CSO.

### Optimization of the Addition Mode and the Weight Ratio of Two *E. Coli* Cells

One addition mode of *Pv*EH1^z4x4-59^ and *Rp*EH^F361V^, in a sequential way, was used for the enantioconvergent hydrolysis of *rac*-*p*CSO (300 mM). In brief, 200 mg wet cells of *E. coli/pveh*1^z4x4-59^ or 40 mg wet cells of *E. coli/rpeh* ^F361V^ was suspended in 1 ml 4% (v/v) Tween-20/phosphate buffer system (100 mM, pH 7.0) described above, while the initial concentration of *rac*-*p*CSO was 300 mM, and the system was incubated at 25°C until (*S*)*-p*CSO or (*R*)*-p*CSO was completely hydrolyzed, then 40 mg wet cells of *E. coli*/*pveh*1^Z4X4-59^ or 200 mg wet cells of *E. coli/pveh*1^z4x4-59^ was added. In a simultaneous way, 200 mg wet cells of *E. coli/pveh*1^z4x4-59^ and 40 mg wet cells of *E. coli/rpeh* ^F361V^ were added at the beginning stage.

Based on the optimized addition mode, the weight ratios of *E. coli/pveh*1^z4x4-59^ to*/rpeh* ^F361V^ from 5:1 to 200:1 were analyzed for the enantioconvergent hydrolysis of *rac*-*p*CSO. In a nutshell, the wet cells weight of *E. coli/pveh*1^z4x4-59^ was 200 mg, and that of *E. coli/rpeh* ^F361V^ was ranging from 40 to 1 mg. The *c* value, *ee*_p_, yield, and the STY (g/L/h) were used as evaluation criteria to get the optimal reaction conditions. STY and the average turnover frequency (aTOF) were calculated by using the following equations: STY(g/L/h) = C_p_/*t*, aTOF(g/g/h) = C_p_/(*t* × C_e_), in which C_p_ was the concentration of (*R*)-*p*CPED (g/L), *t* was the reaction time, and C_e_ was the cell concentration (g/L).

### Gram-Scale Enantioconvergent Hydrolysis of *rac-p*CSO Under the Optimal Reaction Conditions

Using the *c* and *ee*_p_ values as the criteria, the optimal reaction conditions were first confirmed. Subsequently, the scale-up enantioconvergent hydrolysis of *rac*-*p*CSO, in the 100 ml 4% (v/v) Tween-20/phosphate buffer system (100 mM, pH 7.0) containing 200 mg wet cells of *E. coli/pveh*1^z4x4-59^, 40 mg wet cells of *E. coli*/*pveh*1^Z4X4-59^, and 300 mM *rac-p*CSO, was carried out at 25°C. During the hydrolytic process, aliquots of 100 μl samples were drawn out periodically and then analyzed by chiral HPLC until *rac*-*p*CSO was almost completely hydrolyzed (*c* > 99%). In addition, *ee*_p_, yield, and STY were calculated to evaluate its production efficiency. Finally, the aqueous phase was extracted with 20 ml ethyl acetate thrice. The pooled ethyl acetate fractions were washed by saturated NaCl thrice, dried over anhydrous sodium sulfate, and purified by silica gel column chromatography, followed by concentrating under reduced pressure.

## Results and Discussion

### Regioselectivity Assays of *Pv*EH1^Z4X4-59^ and *Rp*EH^F361V^

**FIGURE 1:**
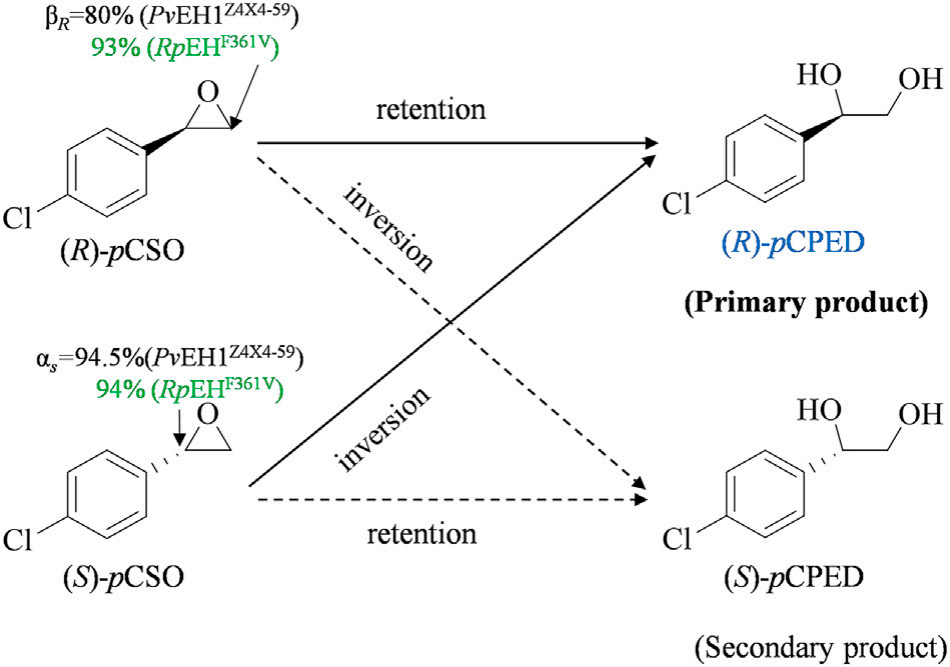
The enantioconvergent hydrolysis of *rac-p*CSO into (*R*)-*p*CPED catalyzed by *E. coli/pveh*1^Z4X4-59^ and *E. coli/rpeh* ^F361V^, respectively. *E. coli/pveh*1^Z4X4-59^ attacks at the C_β_ of (*R*)-*p*CSO with β_*R*_ of 80% mainly affording (*R*)-*p*CPED and at the C_α_ of (*S*)-*p*CSO with α_*S*_ of 94.5% also mainly affording (*R*)-*p*CPED. *E. coli/pveh*1^Z4X4-59^ attacks at the C_β_ of (*R*)-*p*CSO with β_*R*_ of 93% mainly affording (*R*)-*p*CPED and at the C_α_ of (*S*)-*p*CSO with α_*S*_ of 94% also mainly affording (*R*)-*p*CPED.

The regioselectivity coefficients (α_*S*_ and β_*R*_), quantitatively representing its regioselectivities for (*S*)-and (*R*)-*p*CSO, were used to elucidate the *ee*_p_ of (*R*)-*p*CPED produced from the enantioconvergent hydrolysis of *rac*-*p*CSO (Zhang et al., 2020). In our previous study, the kinetic resolution of *rac-p*CSO (800 mM) in the Tween-20/phosphate system buffer by *Pv*EH1^Z4X4-59^ was investigated. The result indicated that *Pv*EH1^Z4X4-59^ enantiopreferentially hydrolyzed the (*S*)-*p*CSO and retained (*R*)-*p*CSO. Herein, to develop the industrial application of *Pv*EH1^Z4X4-59^ in the enantioconvergent hydrolysis of *rac-p*CSO, the regioselectivity coefficients (α_*S*_ and β_*R*_) for *rac-p*CSO were tested. As to a given EH, its regioselectivity coefficients were mainly dependent on the catalyzed *rac*-epoxide, that is, which carbon atom (C_α_ or C_β_) may mainly be subjected to nucleophilic attack by Asp in EH's catalytic triad (Hu et al., 2020a). As shown in Figure 1, the regioselectivity assay of *Pv*EH1^Z4X4-59^ exhibited the hydrolytic reaction of 10 mM (*S*)- and (*R*)-*p*CSO using 200 mg/ml wet cells of *E. coli/pveh*1^z4x4-59^, possessed with 94.5% α_*S*_ and 5.5% β_*S*_ for (*S*)-*p*CSO and 20% α_*R*_ and 80% β_*R*_ for (*R*)-*p*CSO. The reason why *Pv*EH1^Z4X4-59^ can enantiomatically hydrolyze *rac-p*CSO is that it has complementary regionselectivity for (*R*)- and (*S*)-*p*CSO. However, the low β_*R*_ led to the lower final *ee*_p_ of (*R*)-*p*CPED. Contrary to *Pv*EH1^Z4X4-59^, *Rp*EH^F361V^ enantiopreferentially hydrolyzed the (*R*)-*p*CSO, and the enantioselectivity assay exhibited the hydrolytic reaction of 10 mM (*S*)- and (*R*)-*p*CSO using 40 mg/ml wet cells of *E. coli*/*rpeh* ^F361V^, respectively, possessed with 94% α_*S*_ and 6% β_*S*_ for (*S*)-*p*CSO and 7% α_*R*_ and 93% β_*R*_ for (*R*)-*p*CSO. The source of higher enantioconvergence was that it must have high and complementary regioselectivity together with low enantioselectivity. However, there were few wild-type (WT) EHs matching the above conditions simultaneously. Therefore, *Pv*EH1^Z4X4-59^ combined with *Rp*EH^F361V^ may be an ideal solution to hydrolyze *rac-p*CSO for (*R*)-*p*CPED.

### Enantioconvergent of Hydrolysis of *rac-p*CSO by *E. Coli/pveh*1^z4x4-59^ in the Tween-20/Phosphate Buffer System

**FIGURE 2:**
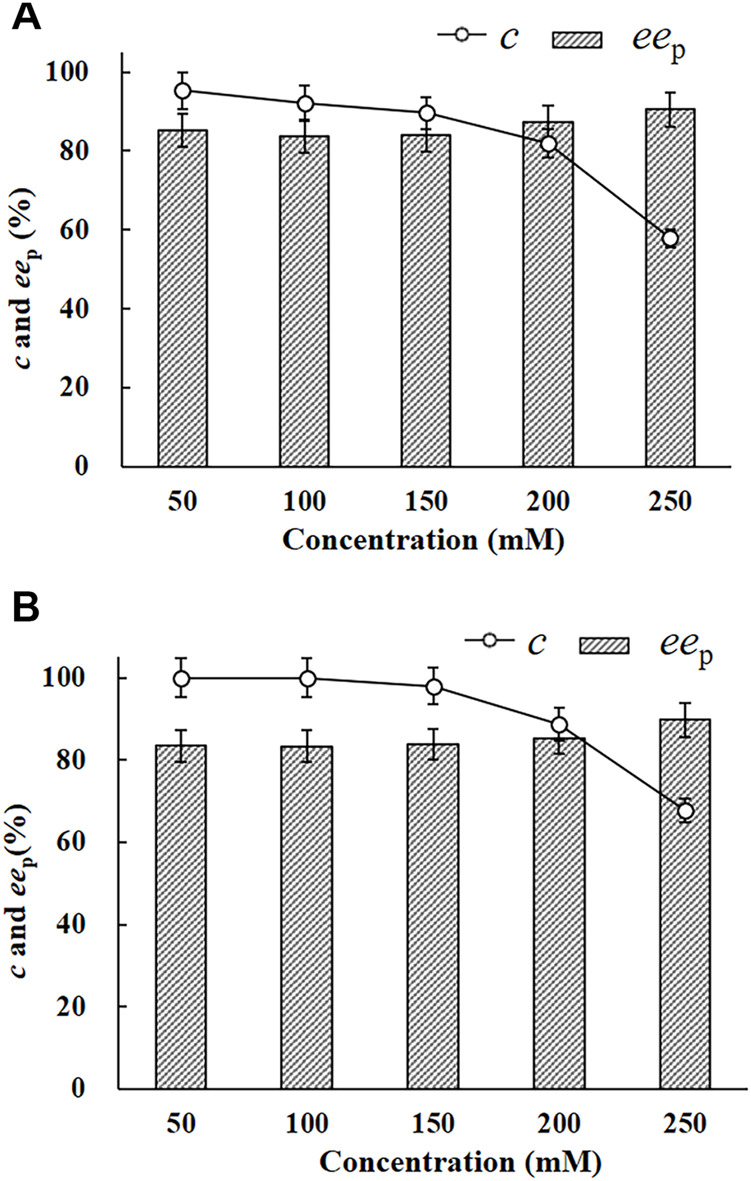
The enantioconvergent hydrolysis of *rac-p*CSO in the 4% (v/v) Tween-20/phosphate buffer system (100 mM, pH 7.0) using 200 mg/ml wet cells of *E. coli*/*pveh*1^z4x4-59^ at 25°C for 12 h **(A)** and 24 h **(B)**. The hydrolytic reactions of *rac-p*CSO at 50–250 mM.

The EH activity of *E. coli*/*pveh*1^z4x4-59^ towards *rac-p*CSO was measured to be 53.2 U/g wet cell, which was 4.2-fold than that of *E. coli/pveh*1 (12.7 U/g wet cell), which was constructed and persevered in our laboratory. No EH activity was detected in the whole cells of *E. coli*/pET-28a (the negative control). Reportedly, the reason for using whole cells instead of crude or purified enzymes as the biocatalyst was the fact that the former was more easily available and usually had higher stability or tolerability, especially at a high substrate and/or product concentration, than the latter during the hydrolytic reaction (Yoo et al., 2008). To investigate the potential for the production of (*R*)-*p*CPED, 200 mg/ml wet cells of *E. coli*/*pveh*1^z4x4-59^ was used to catalyze the enantioconvergent hydrolysis of *rac-p*CSO at different concentrations of 50, 100, 150, 200, and 250 mM in the 4% (v/v) Tween-20/phosphate buffer system (100 mM, pH 7.0). As shown in Figure 2A, 100 mM *rac-p*CSO could not be completely hydrolyzed (92.3% *c*) at 25°C for 12 h, producing (*R*)-*p*CPED with about 83.3% *ee*_p_. When the concentration of *rac-p*CSO was under 100 mM, it was almost completely hydrolyzed at a prolonged hydrolytic time to 24 h (more than 99% *c*). However, when the concentration was elevated to 150 mM, its *c* value was 98% even though the hydrolytic time was prolonged to 24 h (Figure 2B). Consequently, the MAC of *rac-p*CSO was confirmed as 100 mM. This phenomenon was also observed in the enantioconvergent hydrolysis and kinetic resolution of *rac*-epoxides by some other EHs, such as *Ar*EH *and Vr*EH3 (Zou et al., 2013; Hu et al., 2017). This can be explained by a high enantioselectivity (*E* value) towards *rac-p*CSO in the Tween-20/phosphate buffer kinetic resolution for (*S*)-*p*CSO by *E. coli*/*rpeh* ^F361V^, which cannot catalyze the hydrolysis of *rac-p*CSO at a higher concentration in an enantioconvergent way.

### Kinetic Resolution for (*S*)-*p*CSO by *E. Coli*/*rpeh* ^F361V^

**FIGURE 3:**
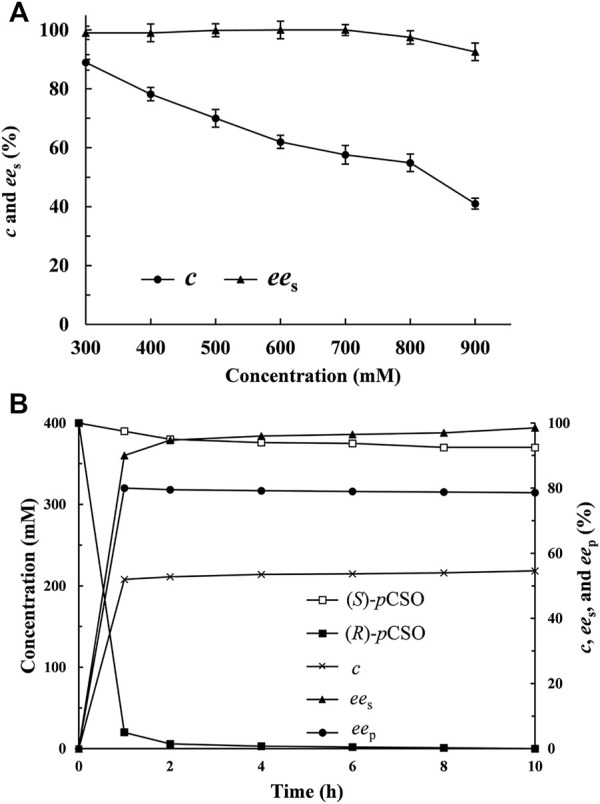
The kinetic resolution of *rac-p*CSO by *E. coli*/*rpeh* ^F361V^. **(A)** the hydrolytic reactions *of rac*-*p*CSO at 300–900 mM in the 4% (v/v) Tween-20/phosphate buffer system (100 mM, pH 7.0) using 40 mg/ml wet cells of *E. coli*/*rpeh* ^F361V^ at 25°C. **(B)** process curves of various parameters in the scale-up kinetic resolution of 800 mM *rac*-*p*CSO at 25°C within 10 h.

Analogously, the enantioselective hydrolytic reactions of *rac-p*CSO at concentrations of 300, 400, 500, 600, 700, 800, and 900 mM were carried out, respectively, at 25°C for 12 h by *E. coli/rpeh* ^F361V^ wet cells. As shown in Figure 3A, the MAC of *rac-p*CSO was confirmed to be 800 mM, which was higher than all those of EHs previously reported, such as 400 mM of *Sl*EH2 and 200 mM of *Sp*EH (Wu et al., 2013; Wen et al., 2021). As the concentration of *rac-p*CSO was elevated to 900 mM, its *c* value and the *ee*_s_ of (*S*)-*p*CSO were merely 40.0% and 72.1% until 12 h. The scale-up kinetic resolution of 800 mM (123.67 g/L) *rac-p*CSO using *E. coli/rpeh* ^F361V^ wet cells was performed in the 30 ml Tween-20/phosphate buffer system and monitored by chiral HPLC at the given intervals (Figure 3B). After incubation for 4 h, (*R*)-*p*CSO was almost completely hydrolyzed at the *c* value of 54.6%, retaining (*S*)-*p*CSO with over 97.2% *ee*_s_ and 45.2% yield. As the hydrolysis of *rac-p*CSO was continued for 10 h, the yield and *ee*_s_ of (*S*)-*p*CSO had no obvious improvement. Consequently, the MAC of *rac-p*CSO was confirmed as 800 mM.

### Enantioconvergent Hydrolysis of *rac-p*CSO by *E. Coli*/*rpeh* ^F361V^ and *E. Coli*/*pveh*1^z4x4-59^

**FIGURE 4:**
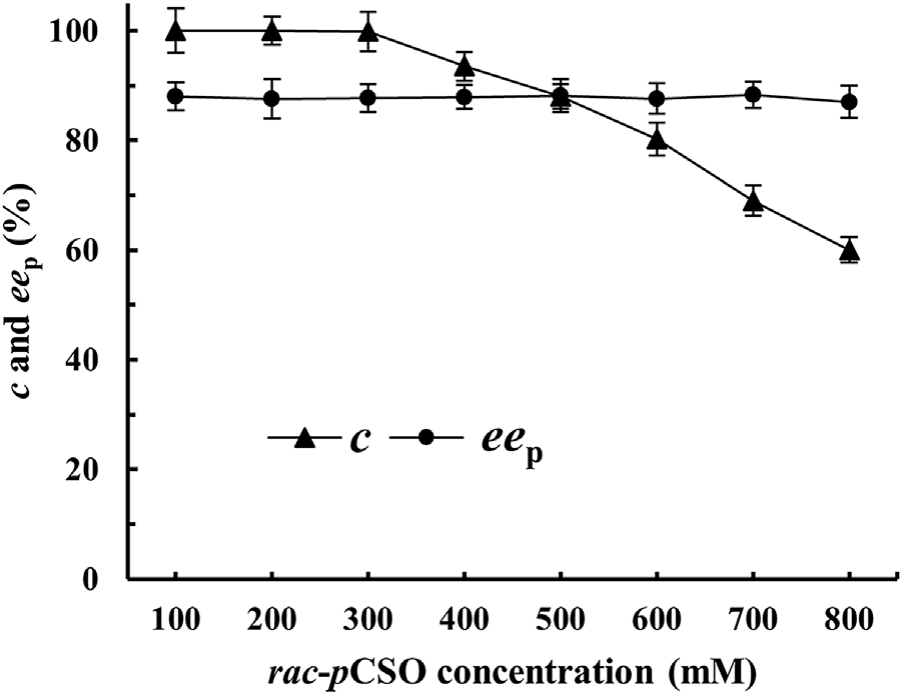
The enantioconvergent hydrolysis of *rac-p*CSO at 100–800 mM in the 4% (v/v) Tween-20/phosphate buffer system (100 mM, pH 7.0) using 200 mg/ml wet cells of *E. coli*/*pveh*1^z4x4-59^ and 40 mg/ml wet cells *E. coli*/*rpeh* ^F361V^ at 25°C for 12 h.

In the above experiment, 100 mM *rac-p*CSO substrate was too low to achieve the industrial production of (*R*)-*p*CPED. To efficiently prepare (*R*)-*p*CPED with high *ee*_p_, yield, and STY, different concentrations of 100–800 mM *rac*-*p*CSO were carried out, respectively, at 25°C using 200 mg/ml wet cells of *E. coli*/*pveh*1^z4x4-59^ and 40 mg/ml wet cells of *E. coli/rpeh* ^F361V^. After incubation for 12 h, 300 mM *rac*-*p*CSO was almost completely hydrolyzed (*c* > 99%), producing (*R*)-*p*CPED with about 87.5% *ee*_p_ (Figure 4). However, when the concentration of *rac*-*p*CSO was increased to 400 mM, its *c* and the *ee*_p_ values were merely 93.5% and 87.9%, which did not meet the requirement of enantioconvergent hydrolysis. Therefore, the MAC was confirmed as 300 mM. In the previous experiments, 800 mM *rac*-*p*CSO could be completely hydrolyzed via the kinetic resolution by *E. coli*/*pveh*1^z4x4-59^ or *E. coli/rpeh* ^F361V^, respectively. Under theoretical conditions, the enantioconvergent hydrolytic maximum concentration of *rac*-*p*CSO by double enzymes should be also at 800 mM. The result indicated that high product concentration rather than substrate concentration affected the activities of *Pv*EH1^Z4X4-59^ and *Rp*EH^F361V^ and that the simultaneous addition of the two enzymes caused a faster reaction speed, producing (*R*)-*p*CPED with high concentration in a short time, which inhibited the activities of the enzymes at the later stage of the reaction. Similarly, in many EH-catalyzed hydrolytic reactions, diol products can inhibit the reaction (Lee and Shuler, 2007).

### Optimization of Both Addition Mode and Weight Ratio of the Two *E. Coli* Cells

**FIGURE 5:**
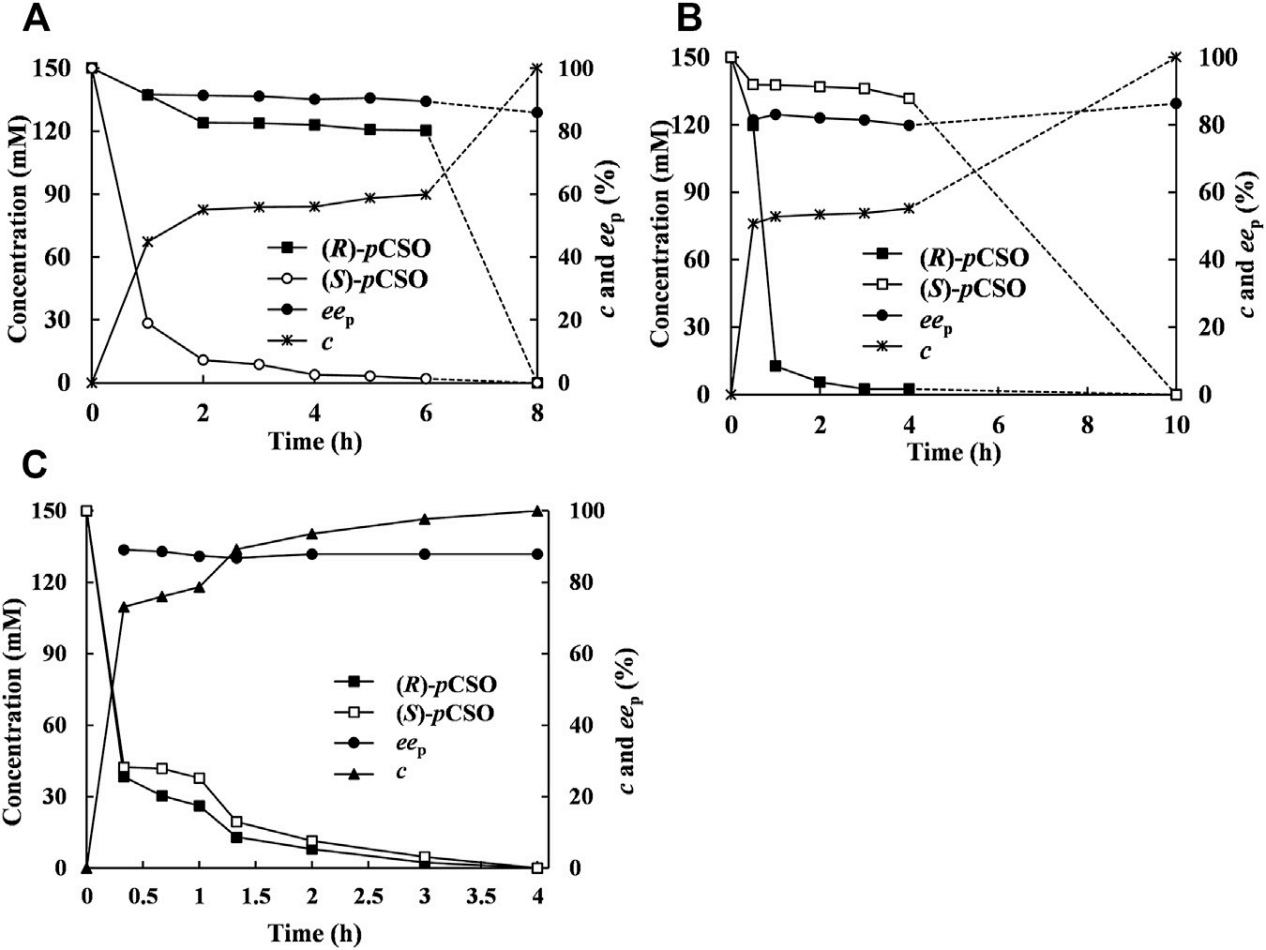
The hydrolytic course curve of *rac-p*CSO catalyzed by *E. coli*/*pveh*1^z4x4-59^ and *E. coli*/*rpeh* ^F361V^. **(A)** bi-enzymatic catalysis in a sequential addition mode. About 300 mM *rac-p*CSO was first hydrolyzed by *E. coli*/*pveh*1^z4x4-59^, and then *E. coli*/*rpeh* ^F361V^ was added to sequentially hydrolyze the residual (*R*)-*p*CSO. **(B)** About 300 mM *rac-p*CSO was first hydrolyzed by *E. coli*/*rpeh* ^F361V^, and then *E. coli*/*pveh*1^z4x4-59^ was added to sequentially hydrolyze the residual (*S*)-*p*CSO. **(C)** Bi-enzymatic catalysis in a simultaneous addition mode. About 300 mM *rac-p*CSO was hydrolyzed by adding both *E. coli*/*rpeh* ^F361V^ and *E. coli*/*pveh*1^z4x4-59^ simultaneously. The enantioconvergent hydrolysis of *rac-p*CSO was in the 4% (v/v) Tween-20/phosphate buffer system (100 mM, pH 7.0) at 25°C.

In addition to the selected Tween-20/phosphate buffer system towards the given pair of EH-substrate, the addition mode may be an important parameter influencing the enzymatic catalytic performance (Wang et al., 2017). In the sequential addition mode, 300 mM *rac*-*p*CSO was first hydrolyzed by 200 mg/ml wet cells of *E. coli*/*pveh*1^z4x4-59^ for 6 h until (*S*)-*p*CSO was completely hydrolyzed, retaining (*R*)-*p*CSO with 59.9% *c* and 96.8% *ee*_s_. Then, 40 mg/ml wet cells of *E. coli/rpeh* ^F361V^ was added to sequentially hydrolyze the residual (*R*)-*p*CSO for 2 h, thereby affording (*R*)-*p*CPED with 85.8% *ee*_p_, 91.5% yield, and 5.32 g/L/h STY at 100% *c* (Figure 5A). This result indicated that, by adding an extra complementary *Rp*EH^F361V^, the *ee*_p_ of (*R*)-*p*CPED was slightly increased from 83.3% to 85.8%, while the reaction time was significantly shortened from 24 to 8 h and the substrate concentration was increased from 100 to 300 mM. Similarly, 300 mM *rac*-*p*CSO was first hydrolyzed by 40 mg/ml wet cells of *E. coli/rpeh* ^F361V^ for 4 h, retaining (*S*)-*p*CSO with 55.2% *c* and 96.5% *ee*_s_. Then, 200 mg/ml wet cells of *E. coli*/*pveh*1^z4x4-59^ was added to sequentially hydrolyze the residual (*S*)-*p*CSO for 6 h, thereby affording (*R*)-*p*CPED with 86.3% *ee*_p_, 92.1% yield, and 4.28 g/L/h STY at 100% *c* (Figure 5B). In another addition mode, 300 mM *rac*-*p*CSO was hydrolyzed for 4 h by adding *E. coli*/*pveh*1^z4x4-59^ and *E. coli/rpeh* ^F361V^ simultaneously, affording (*R*)-*p*CPED with 87.5% *ee*_p_, 93.2% yield and 10.8 g/L/h STY at 100% *c* (Figure 5C). Compared with the sequential addition mode, the STY of the simultaneous addition mode was significantly improved, while *ee*_p_ and yield of (*R*)-*p*CPED showed no significant difference. Although 800 mM *rac*-*p*CSO can be completely hydrolyzed by kinetic resolution, the reaction time could be up to 12 h, and the concentration (360 mM) of the product near the end point of the reaction may inhibit the enzymatic activities. Therefore, the simultaneous addition mode could cause a faster reaction speed and produce (*R*)-*p*CPED with a high concentration in a short time, which did not affect the enzymatic activities during the rapid reaction phase. This result was similar to that reported previously, such as using 150 U *St*EH and 500 U *An*EH in 125 ml buffer solution in a sequential addition mode, *rac*-pCSO can be completely hydrolyzed with about 2 days, affording (*R*)-*p*CPED with 96% *ee*_p_ and 93% yield (Manoj et al., 2001). Consequently, the simultaneous addition mode was selected to save time and improve production efficiency.

The *ee*_p_ of (*R*)-*p*CPED was increased from 82.2% to 87.8% as the weight ratios of *E. coli*/*pveh*1^z4x4-59^ and *E. coli/rpeh* ^F361V^ range from 200:1 to 20:1 while slightly decreased to 87.5% as the weight ratio increased to 200:40 (Table 1). Consequently, considering the highest *c* value, *ee*_p_, and yield, the weight ratio was confirmed as 20:1. The weight ratio of two whole cells or enzymes for the bi-enzymatic catalysis had a distinct effect on the *ee*_p_ of enantioconvergent hydrolysis of racemic epoxides. Enantioconvergent hydrolysis of *rac*-SO was achieved by using two recombinant epoxide hydrolases of *Cc*EH and *Mc*EH, affording (*R*)-PED with 92% *ee*_p_ at the optimized weight ratio of 0.3:1, which was higher than that (89% *ee*_p_) at the ratio of 0.1:1 (Kim et al., 2008).

**TABLE 1:** The enantioconvergent hydrolysis of *rac-p*CSO at elevated weight ratios in the 4% (v/v) Tween-20/phosphate buffer system.

*Pv*EH1^Z4X4-59^	*Rp*EH^F361V^	Time	*c*	*ee*_p_	Yield
(mg/ml)	(mg/ml)	(h)	(%)	(%)	(%)
200	40	4	100	87.5	93.2
200	20	5	100	87.7	93.3
200	10	5	100	87.8	93.4
200	5	8	97.6	87	91.3
200	1	10	93.3	82.2	84.9

### Gram-Scale Production of (*R*)-*p*CPED via Enantioconvergent Hydrolysis of *rac-p*CSO

**FIGURE 6:**
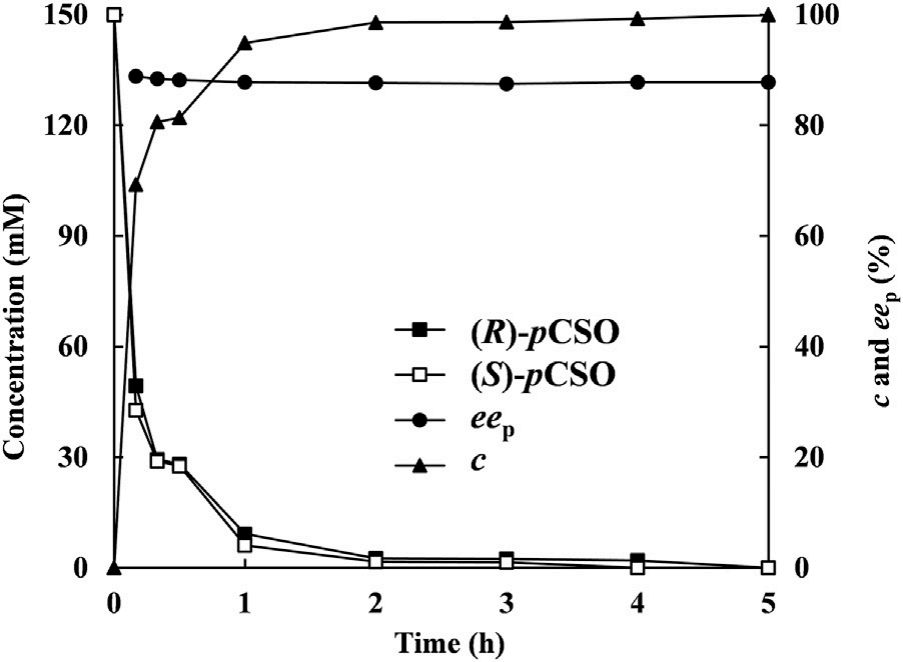
The hydrolysis of 300 mM *rac-p*CSO in the 4% (v/v) Tween-20/phosphate buffer system (100 mM, pH 7.0) by *E. coli*/*rpeh* ^F361V^ and *E. coli*/*pveh*1^z4x4-59^ under the optimized conditions—the time courses of (*R*)- and (*S*)-*p*CSO concentrations, *c* of *rac-p*CSO and *ee*_p_ of (*R*)-PED.

Under the above optimized reaction conditions, the enantioconvergent hydrolysis of *rac-p*CSO was conducted in the 100 ml 4% (v/v) Tween-20/phosphate buffer system (100 mM, pH 7.0) and monitored by HPLC at given time intervals (Figure 6). After incubation for 5 h, 300 mM *rac-p*CSO was completely hydrolyzed, affording (*R*)-*p*CPED with 87.8% *ee*_p_, 93.4% yield, and 8.63 g/L/h STY. Subsequently, 4.20 g (*R*)-*p*CPED was produced in 81.1% isolated yield after being purified by silica gel column chromatography. The enantioconvergent hydrolysis of *rac-p*CSO by using two *E. coli* cells expressing *Pv*EH1^z4x4-59^ and *Rp*EH^F361V^ exhibited the highest substrate concentration and STY (300 mM and 8.63 g/L/h) among all known enantioconvergent hydrolytic reactions, such as those by the combinations of *St*EH and *An*EH (4 mM, 0.24 g/L/h, and 89% *ee*_p_) (Karboune et al., 2005), an *E. coli* transformant expressing *Caulobacter crescentus* EH (109 mM, 4.29 g/L/h and 95% *ee*_p_) (Hwang et al., 2008), and a double-site mutant *E. coli/pveh3* ^G170E/F187X^ (150 mM, 1.50 g/L/h, and 92.8% *ee*_p_) (Hu et al., 2018). Although the substrate concentration and STY were higher, the *ee*_p_ value was a little low. In the following work, protein engineering methods will be used to improve *ee*_p_ value.

## Conclusion

Enantioconvergent hydrolysis of *rac-p*CSO by *Pv*EH1^Z4X4-59^ and kinetic resolution for (*S*)-*p*CSO by *Rp*EH^F361V^ were investigated at elevated concentrations. Based on their high and complementary regioselectivities, a bi-enzymatic catalysis technique using two recombinant *E. coli* wet cells was designed and optimized for the enantioconvergent hydrolysis of *rac-p*CSO at high concentration. Both addition mode and weight ratio of the two *E. coli* cells were optimized. A simultaneous addition mode of both *E. coli*/*pveh*1^z4x4-59^ and *E. coli/rpeh* ^F361V^ was first selected, by which the STY was significantly improved. Then, considering the highest *ee*_p_ and aTOF values, the weight ratio was confirmed as 20:1. Finally, in the 100 ml 4% (v/v) Tween-20/phosphate buffer system (100 mM, pH 7.0) under the optimized weight ratio and the simultaneous addition mode, the enantioconvergent hydrolysis of *rac-p*CSO at 300 mM was carried out, producing (*R*)-*p*CPED with 87.8% *ee*_p_, 93.4% yield, and 8.63 g/L/h STY. In conclusion, this work would provide an efficient technical strategy for the preparation of chiral vicinal diols at industrial scale.

## Data Availability

The original contributions presented in the study are included in the article/Supplementary Material, further inquiries can be directed to the corresponding author.
